# Concurrent Parasitic Infections in a Renal Transplant Patient

**DOI:** 10.3201/eid1912.120926

**Published:** 2013-12

**Authors:** Govinda S. Visvesvara, Michael J. Arrowood, Yvonne Qvarnstrom, Rama Sriram, Rebecca Bandea, Patricia P. Wilkins, Eileen Farnon, Gill Weitzman

**Affiliations:** Centers for Disease Control and Prevention, Atlanta, Georgia, USA (G.S. Visvesvara, M.J. Arrowood, Y. Qvarnstrom, R. Sriram, R. Bandea, P.P. Wilkins, E. Farnon);; NewYork–Presbyterian/Weill Cornell Medical Center, New York, New York, USA (G. Weitzman)

**Keywords:** cyclospora, enterocytozoon cystoisospora, microsporidia, protozoa, parasitic, parasite, fungi, kidney, renal, transplant, gram chromotrope, diarrhea, albendazole, trimethoprim, sulfamethoxazole

**To the Editor:** Protozoan pathogens, including *Entamoeba histolytica*, *Giardia*, *Cryptosporidium*, *Cyclospora*, *Cystoisospora*, and microsporidia such as *Enterocytozoon bieneusi*, are well-known agents of diarrhea and a major public health problem in developing countries. Infection with *Cyclospora cayetanensis* and *E. bieneusi* can occur in immunocompromised and immunocompetent persons. Severe diarrhea and weight loss along with anorexia, nausea, and low-grade fever occur in immunocompromised persons, particularly those with HIV/AIDS and transplant recipients who are taking immunosuppressive drugs (*1*,*2*). However, transient diarrhea occurs in immunocompetent persons, notably in travelers returning from countries with poor hygienic standards (*1*–*3*).

We report on a kidney transplant recipient who had uncontrollable diarrhea and weight loss in whom *C. cayetanensis* and *E. bieneusi* were detected in biopsy specimens; the diarrhea resolved after treatment with drugs that act specifically on these 2 parasites. The patient was a 55-year-old man from the Dominican Republic living in New York, NY, USA; he had a history of long-term diabetes, coronary disease, and alcoholism. He had undergone a cadaveric renal transplant 14 months earlier and had an uneventful posttransplant course. After returning from visiting family in the Dominican Republic, he sought treatment for acute, profuse watery diarrhea in early November, 2009. He had >10 watery bowel movements daily that were associated with a 20-lb weight loss. His symptoms persisted for 2 months, and he required 2 hospitalizations for the diarrhea. 

Results of 4 repeat fecal specimen tests (routine diagnostic microscopy and culture) were negative for parasites. Colonoscopy findings were normal; because of evidence of leukocytes in the feces and elevated fecal fat level, however, he received empirically prescribed metronidazole. Because his diarrhea and weight loss persisted, an upper endoscopy was performed, which revealed the presence of microsporidia. He then received albendazole for 3 weeks without substantial benefit. 

The biopsy specimens were sent to the Centers for Disease Control and Prevention (Atlanta, GA, USA) for further analysis. Biopsy slides were stained with hematoxylin and eosin and with Gram chromotrope (*4*) and examined by microscopy. The Gram chromotrope–stained slide revealed oval spores, pinkish-red in color, measuring ≈1 µm (*5*). These spores were supra nuclear in position and were consistent with *E. bieneusi* (Figure, panel A). The tissue sections were scraped from the slides, DNA was extracted, and conventional PCR was performed by using *E. bieneusi*–specific primers as described (*5*); the sizes of the amplified product in the tissue DNA specimen and in the *E. bieneusi* control specimen were identical (Figure, panel B), confirming the presence of *E. bieneusi*. On further microscopic examination of the Gram chromotrope and the hematoxylin and eosin–stained slides, oval bodies (8–10 µm) were seen. A few of these oval bodies exhibited 4 spindle-shaped structures which were identified provisionally as merozoites of a coccidian parasite (Figure, panel C). Others had morula-like internal structure (Figure, panel D). We hypothesized that the coccidian parasite could either be *C. cayetanensis* or *Cystoisospora hominis*. Because the parasites, in various stages, were just beneath the surface of the epithelium, rather than deep within the epithelium, we believed this organism to be a *Cyclospora* sp. rather than a *Cystoisospora* sp. We searched the serum bank of the Division of Parasitic Diseases, Centers for Disease Control and Prevention, and identified a serum sample from a person with a case of *C. cayetanensis* cyclosporiasis. An indirect immunofluorescence test was performed by using this serum on a deparaffinized section of the tissue biopsy specimen. Different stages of the coccidian organism were labeled brightly and produced apple-green fluorescence against a red counterstain (Eriochrome Black T), indicating that the parasite could possibly be a *Cyclospora* sp. (Figure, panels E, F). We considered that the *Cyclospora*-positive serum sample obtained from this particular patient may not be species-specific, since he might have also been infected with *Cystoisospora*. Therefore, we performed a real-time PCR assay that can distinguish *C. cayetanensis* from other coccidian parasites to identify the parasite definitively (*3*). DNA recovered from tissue in paraffin sections was successfully amplified and detected with this assay (data not shown), confirming the presence of *C. cayetanensis*.

**Figure:**
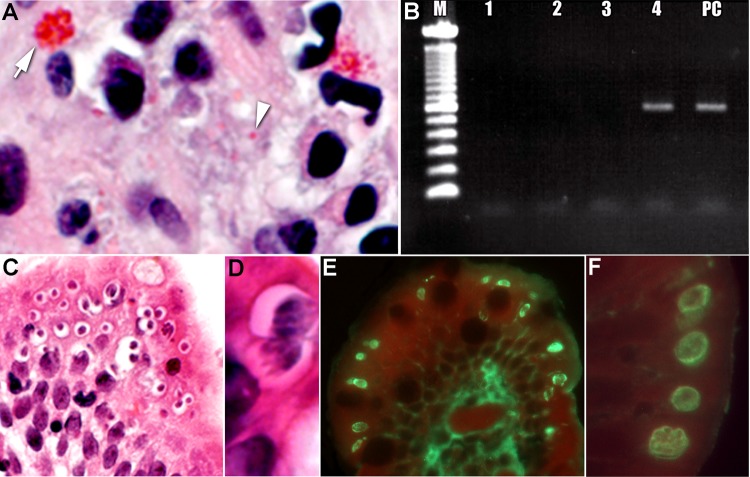
Tissue specimens from a kidney transplant recipient with concurrent parasitic infections after traveling to the Dominican Republic. A) Tissue section stained with Gram chromotrope. Note the apical location of a cluster of *Enterocytozoon bieneusi* spores at arrow (original magnification ×1,000) and single spore at arrowhead.; B) Agarose gel showing PCR amplification of *E. bieneusi* 18S rDNA in the scraped section, as in panel A (M, 100-bp ladder; lane 1, DNA lysate diluted 1:5; lane 2, 1:10, lane 3, 1: 50 and lane 4, 1:100 of DNA lysate; lane 5 PC, positive control specimen). C) Tissue section stained with hematoxylin and eosin, demonstrating numerous sites in which *Cyclospora* spores are in developing stages (original magnification ×100). D) Higher power image of *Cyclospora* spores, showing the developing meronts (original magnification ×1,200). E) Immunofluorescent reactivity (bright green) of the various life cycle stages of *Cyclospora* with a positive anti-*Cyclospora* serum sample (original magnification ×200). F) Note the bright fluorescence of the various parasite stages just below the apical (luminal) surface of the epithelial cells (original magnification ×1,000).

The patient’s illness was treated with albendazole for *E. bieneusi* infection and with trimethoprim and sulfamethoxazole for *C. cayetanensis* infection. The patient’s diarrhea subsided after 1 week, and several subsequent fecal samples were negative for microsporidia spores and *Cyclospora* oocysts. His immunosuppressive medications were reduced, and he remained diarrhea-free for the following 3-year period of April 2010 to April 2013. 
